# Amantadine Inhibits SARS-CoV-2 In Vitro

**DOI:** 10.3390/v13040539

**Published:** 2021-03-24

**Authors:** Klaus Fink, Andreas Nitsche, Markus Neumann, Marica Grossegesse, Karl-Heinz Eisele, Wojciech Danysz

**Affiliations:** 1 Merz Pharmaceuticals GmbH, 60318 Frankfurt, Germany; Karl-Heinz.Eisele@merz.de (K.-H.E.); Wojciech.Danysz@Merz.de (W.D.); 2 Robert-Koch-Institut, Zentrum für Biologische Gefahren und Spezielle Pathogene: Hochpathogene Viren (ZBS 1), 13353 Berlin, Germany; NitscheA@rki.de (A.N.); NeumannM@rki.de (M.N.); GrossegesseM@rki.de (M.G.)

**Keywords:** amantadine, antiviral drugs, Covid-19, SARS-CoV-2

## Abstract

Since the SARS-CoV-2 pandemic started in late 2019, the search for protective vaccines and for drug treatments has become mandatory to fight the global health emergency. Travel restrictions, social distancing, and face masks are suitable counter measures, but may not bring the pandemic under control because people will inadvertently or at a certain degree of restriction severity or duration become incompliant with the regulations. Even if vaccines are approved, the need for antiviral agents against SARS-CoV-2 will persist. However, unequivocal evidence for efficacy against SARS-CoV-2 has not been demonstrated for any of the repurposed antiviral drugs so far. Amantadine was approved as an antiviral drug against influenza A, and antiviral activity against SARS-CoV-2 has been reasoned by analogy but without data. We tested the efficacy of amantadine in vitro in Vero E6 cells infected with SARS-CoV-2. Indeed, amantadine inhibited SARS-CoV-2 replication in two separate experiments with IC_50_ concentrations between 83 and 119 µM. Although these IC_50_ concentrations are above therapeutic amantadine levels after systemic administration, topical administration by inhalation or intranasal instillation may result in sufficient amantadine concentration in the airway epithelium without high systemic exposure. However, further studies in other models are needed to prove this hypothesis.

## 1. Introduction

Antiviral drugs have been quite successful treatments of Influenza A, Cytomegalovirus, Herpes Simplex and Herpes Zoster Viruses, Human Immunodeficiency Virus, Hepatitis B and C, and Respiratory Syncytial Virus or Human Papilloma Virus infections [1]. This is a remarkable success because the pharmacotherapeutic strategy behind is more difficult than for antibacterial drugs. This is because viruses hijack and reprogram cells to produce new virus components and finally release new virus particles, whereas bacteria mostly live and reproduce on their own. As a consequence, antiviral drugs need to inhibit virus entry into, prohibit virus replication within, or prohibit virus release out of the host cell while preserving the host cell. In contrast, antibacterial drugs destroy the bacteria itself or interfere with bacterial metabolism or life cycle steps [2]. For COVID-19 treatment, all available options are considered, including vaccines, antiviral drugs, and antiviral antibodies. Some vaccines have just been approved or are far advanced in terms of development because pre-existing tool boxes could be used for the design. Specific antiviral drugs are not so far developed because complete drug development would take 7–10 years as a rule. Although some monoclonal antibodies directed against the receptor binding site of the SARS-CoV-2 spike protein have also been developed extremely fast [3,4,5], many pharmaceutical companies have repurposed antiviral drugs to shorten the time frame, with limited success. In parallel, research on the COVID-19 disease process has presented other treatment options such as dexamethasone. Besides these evidence-based approaches a multitude of drugs has been considered as a potential cure for COVID-19 [6]. Amantadine was approved in 1966 in Germany for the prophylaxis of Asian influenza and in 1976 for the treatment of Influenza A. Amantadine is also approved for Parkinson’s disease. Other repurposed antiviral compounds such as remdesivir or hydroxychloroquine that were in vitro effective against SARS-CoV-2 turned out to have little or no effect against COVID-19 in vivo [7]. Antiviral drugs against SARS-CoV-2 will remain valuable treatment options for COVID-19 because SARS-CoV-2 infections will not immediately disappear as soon as larger parts of the global population get vaccinated. It is even conceivable that patients may suffer from COVID-19 disease although they were vaccinated.

Coronaviruses use peptidases as entry receptors, e.g., Angiotensin Converting Enzyme 2 (ACE2), by SARS-CoV and HCoV-NL63 or dipeptidyl peptidase 4 (DPP4) by MERS-CoV [8]. The SARS-CoV-2 mode of infection is initiated by binding of the SARS-CoV-2 spike protein (S) to ACE2, after host cell priming of S by the serine protease TMPRSS2 [9]. Amantadine exerts several pharmacodynamic effects [10] from which inhibition of the influenza A M2 ion channel is relevant for its antiviral efficacy against influenza A [11,12]. It is also used for the treatment of Parkinson’s Disease due to its low-potent noncompetitive inhibition of the NMDA receptor ion channel on striatal dopaminergic neurons [13]. The viral M2 protein is considered a viroporin that forms a cation-selective ion channel that may be involved in influenza A virus uptake into the host cell [14,15]. Although there is no sequence homology of the influenza A and SARS-CoV-2, amantadine inhibits two of the four known (protein 3a and E) or putative (Orf7b and Orf10) SARS-CoV-2 viroporins, protein E and Orf10a [16,17]. SARS-CoV E protein forms a transmembrane channel [18,19,20]. Substantial research on SARS-CoV was performed after the SARS-CoV pandemic in 2003, and many findings may be transferred to SARS-CoV-2. Both viruses share 79% genomic sequence [21]; the E protein shares even 94% of the sequence, and its ion-conducting transmembrane domain is identical [16]. The corona virus E proteins form a homopentameric cation channel [18,20,22,23] that is involved in the induction of apoptosis, membrane permeability, and inflammation [24,25], and represents a potential drug target.

Amantadine, which inhibits several ion channels such as NMDA and M2, is suspected to also inhibit Coronavirus ion channels. It was approved by the US Food and Drug Administration (FDA) for the treatment of influenza A in 1976 and was used as such until point mutations in the influenza A genome resulted in increasing resistance; CDC recommended in 2009 not to use it for influenza A anymore. In fact, the 2008/2009 seasonal influenza (H3N2) and the 2009 swine flu pandemic (H1N1) were almost completely resistant to amantadine [26]. It was the objective of the present study to elucidate the antiviral efficacy of amantadine against SARS-CoV-2 in vitro and compare it to the existing human safety data in order to provide a reasonable assessment of its feasibility for COVID-19 therapy.

Remdesivir, also a repurposed antiviral drug, is a nucleoside triphosphate analog that inhibits the RNA-dependent RNA polymerase of some RNA viruses. While the in vitro antiviral pharmacodynamics for SARS-CoV-2 have been convincingly shown [27], the clinical efficacy against COVID-19 is still disputed. Based on the ACTT-1 study [28], the European Medicines Agency (EMA) granted a conditional marketing authorization for remdesivir for patients suffering from COVID 19 pneumonia with oxygen supplementation in July 2020. The FDA approved it in October 2020 for the treatment of COVID-19, although the WHO Solidarity trial [29] could neither demonstrate a therapeutic effect of remdesivir nor of further three repurposed drugs hydroxychloroquine, lopinavir, and interferon β1a. In this study, remdesivir is used as a comparator, in the best case as a positive control, with proven in vitro antiviral efficacy against SARS-CoV-2.

## 2. Materials and Methods

### 2.1. Test Compounds

1-amino adamantane hydrochloride (Amantadine HCl, Sigma-Aldrich, St. Louis, MO, USA), remdesivir (Cayman Chemicals, Ann Arbor, MI, USA, No. 30345), and camostat mesylate (Sigma, St. Louis, MO, USA, SML0057) were dissolved in DMSO at 10 mM final concentration. Stock solutions were stored at −20 °C.

### 2.2. S-Protein—ACE2 Binding Assay

The compounds were tested for their ability to inhibit the binding of SARS-CoV-2 spike protein (S protein) to ACE2 using the SARS-CoV-2 spike: ACE2 Inhibitor Screening Assay Kit (BPS Bioscience #79931, San Diego, CA, USA). In brief, the SARS-CoV-2 spike protein was coated to a 96 microwell plate at 1 µg/mL in phosphate buffered saline. Unbound protein was removed and unspecific binding sites in the wells are blocked. Then, the blocking solution was removed, and the diluted compounds and control samples were added to the wells. After pre-incubation of the coated spike protein with the compounds, the His-tagged ACE2 protein was added and incubated together with the compounds to allow binding to the spike protein. After washing and blocking, the bound ACE2 protein was detected by an anti-His-antibody coupled to horse radish peroxidase (HRP). The detection was performed using a chemiluminescent HRP substrate and reading the luminescence intensity in a microtiter-plate reader. The luminescence signal of each sample containing diluted compound was divided by the luminescence in absence of any inhibitor, and the resulting values were plotted against the concentration of the compound.

### 2.3. Cell Line

Cytotoxicity and antiviral activity of the drugs were studied in Vero E6 cells (Cercopithecus aethiops, kidney, ECACC #85020206 for 1st experiment and ATCC CRL-1586 for 2nd experiment). The cell line is routinely maintained in DMEM supplemented with 2 mM glutamine, 5% fetal bovine serum at 37 °C, and 5% CO2 in a humified atmosphere, and can be infected with SARS-CoV-2 [30].

### 2.4. Antiviral Activity Assay with RT-PCR Readout (1st Experiment)

Exponentially growing Vero E6 cells were seeded into a 48-well plate at a density of 8 × 10^4^ cells per well and were incubated overnight. Medium was removed and cells were infected in triplicate with SARS-CoV-2 (hCoV-19/Italy/INMI1-isl/2020 (National Institute for Infectious Diseases, Rome, Italy, GISAID Accession EPI_ISL_410545) at an MOI of 0.01 in 300 µL of medium containing different inhibitor concentrations. Amantadine was solubilized in sterile water and further diluted with medium to concentrations of 500 µM, 100 µM, 20 µM, 4 µM, and 8 nM. Remdesivir [31] was solubilized in DMSO and diluted with medium to concentrations of 50 µM, 10 µM, 2 µM, 0.4 µM, and 80 nM. Remdesivir MOCK control contained according amounts of DMSO.

Cells were washed with PBS at 2 h post infection (p.i.) and 400 µL of fresh medium containing the respective concentration of the antiviral substances were added. The amount of SARS-CoV-2 RNA in the supernatant and cells was analyzed 24 h p.i. by qPCR.

Briefly, 180 µL of supernatant was taken and stored at −80 °C prior to extraction. After complete removal of supernatant, cells were washed with 500 µL of PBS and lysed for 5 min at RT with 350 µL of RNeasy lysis buffer (RLT buffer, Qiagen, Hilden, Germany) containing 1% 2-mercaptoethanol. Lysates were transferred into microcentrifuge tubes, inactivated for 1 h at 60 °C and extracted with the RNeasy Kit (Qiagen) according to manufacturer’s instructions.

Supernatants were extracted with internal KoMa control and subjected to qPCRs targeting the E gene. Inhibition was calculated by normalization of SARS nucleic acids to KoMa (supernatant) or MYC transcripts (lysed cells, myc-gene refers to myelocytomatosis) referred to the infected control without treatment. To guarantee RNA-specificity of the MYC assay the following primers and probe were used: MYC F: gggTAgTggAAAACCAgCCT; MYC R: TCgTCgCAgTAgAAATACgg; MYC probe Cy5-TATgACCTCgACTACgACTCggTgC-BHQ1. The half maximal inhibitory concentration (IC_50_) values for each inhibitor were by calculated using nonlinear regression analyses with Graph Pad Prism 8.40 software (GraphPad Software, San Diego, CA, USA).

Cell viability was determined with the ATP-based CellTiter-Glo® luminescent cell viability assay (Promega, Madison, WI, USA) 26 h after antiviral substance addition. Viability of uninfected Vero E6 cell cultures treated with amantadine and remdesivir was analyzed in at least three biological replicates according to the manufacturer’s instruction with an adapted homogenization time on shaker of 15 min for efficient lysis. After incubation, 200 µL of lysed sample was diluted with 220 µL of fresh medium. For measurement, technical duplicates of each sample with an amount of 200 µL were transferred into a 96-well white polystyrene microplate (Corning, Corning, NY, USA). Luminescence was measured in an Infinite plate reader with i-control software (Tecan, Männedorf, Switzerland), and viability was calculated referred to untreated controls.

### 2.5. Antiviral Activity Assay with Nucleocapsid Protein Readout (2nd Experiment)

Exponentially growing Vero E6 cells were seeded into a 96-well plate at their optimal density in complete medium; 24 h later, cells were infected with SARS-CoV-2 (viral strain INMI1) at 0.01 moi (multiplicity of infection) and then exposed to different concentrations of the drugs (0–0.1–1–10–100–300 μM for amantadine) for 72 h. Drug dilutions were performed in culture medium. Replicates for each concentration point were examined. At the end of the incubation period, antiviral activity was examined through both ELISA (Sino Biological, quantifying SARS-CoV-2 nucleoprotein) as well as a cytoprotection assay (toxicity effect examined through an inverted microscope).

### 2.6. Data Analysis

The results are given as means ± SD of n experiments. If appropriate, IC_50_ values were calculated from a sigmoidal logistic equation fitted to the concentration-response data by iterative nonlinear regression analysis (Prism; GraphPad Software Inc., San Diego, CA, USA). For comparison of mean values, the two-tailed *t*-test (for unpaired data) or Welch’s *t*-test (in case of unequal variances) was used. The minimal level of significance was *p* < 0.05.

## 3. Results

**Figure 1:**
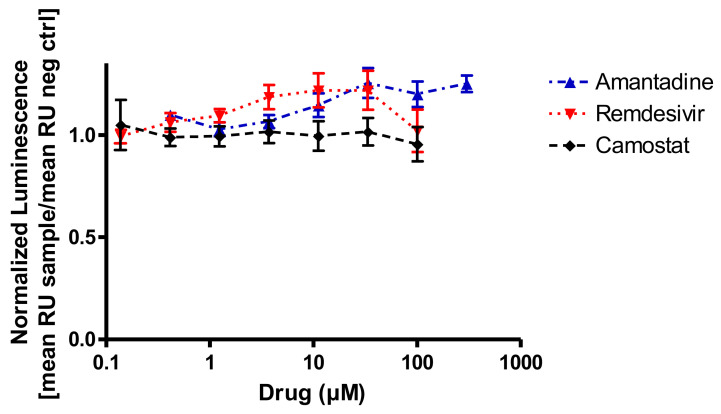
In vitro binding interference of the SARS-CoV-2 spike protein to recombinant ACE2 and amantadine, remdesivir, or camostat mesylate. None of the drugs inhibit SARS-CoV-2 binding to its target protein (RU, relative units; n = 3 assays in duplicate).

As it is unclear which type of interference amantadine might have with SARS-CoV-2 infection or replication, the first step of the viral infection was addressed, which is the binding of the viral spike protein to ACE2 on target cells. We chose a competitive binding assay in an ELISA format in order to detect potential inhibitory effects. Amantadine (0.3–300 µM) did not inhibit the binding of the SARS-CoV-2 spike protein to human ACE2 protein (Figure 1). One might rather speculate on a numerical increase in binding at high concentrations above 10 µM but given the low number of replicates it should be considered as neither significant nor relevant. Remdesivir was tested in this system because it is not supposed to inhibit binding of the spike protein but to affect viral replication. Camostat mesylate was included in this assay because it is supposed to affect virus entry into the target cell, not by inhibiting spike protein binding to ACE2 but by inhibiting the downstream human serine protease TMPRSS2 activation [32]. TMPRSS2 cleaves the spike protein, which is a requirement for the plasma membrane fusion pathway for SARS-CoV-2 entry. Thus, it was not expected that camostat mesylate would inhibit binding of S-protein to ACE2. Neither remdesivir (0.1–100 µM) nor camostat mesylate (0.1–100 µM) had any effect on the S-protein binding to ACE2 in this test paradigm (Figure 1).

**Figure 2:**
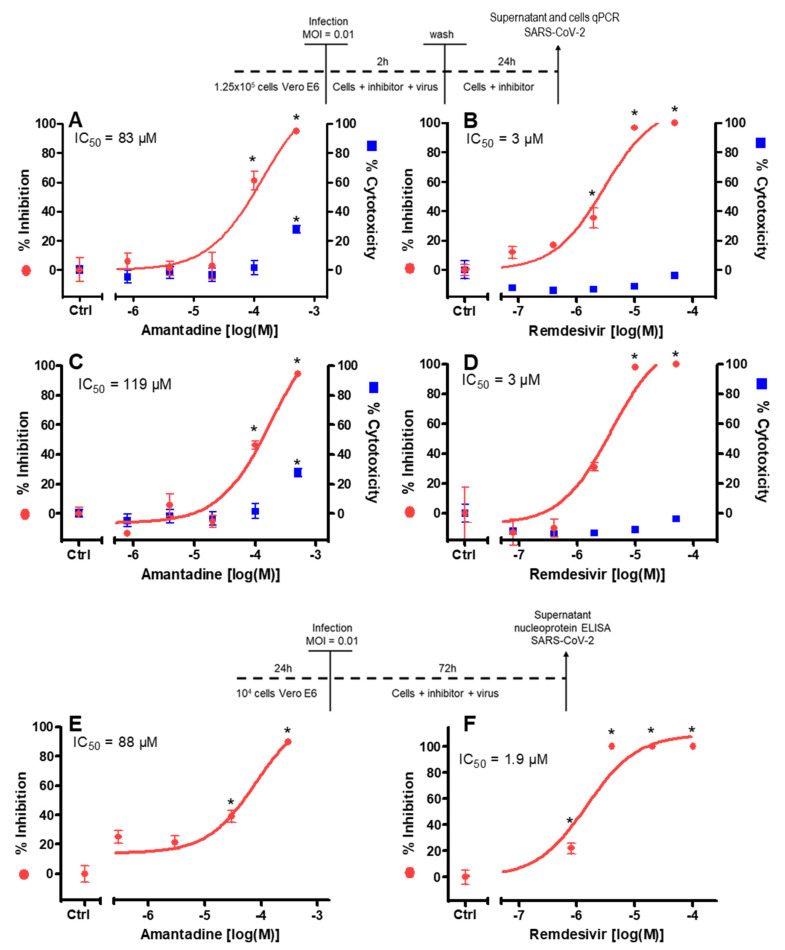
Effect of amantadine on SARS-CoV-2 replication in vitro in the first (**A**–**D**) and second (**E**,**F**) series of experiments. Vero E6 cells were treated with amantadine and infected according to the displayed schedules. Remdesivir was used as a positive control. (**A**–**D**) 26 h after infection and inhibitor addition, the virus yield in the supernatant (**A**,**B**) was quantified by qPCR (left y-axis, red symbols) and the cytotoxicity was analyzed by CTG assay (CellTiter-Glo^®^ viability assay; right y-axis, blue symbols). In addition, the virus yield in the cells (**C**,**D**) was quantified by qPCR. (**E**,**F**) The virus nucleocapsid protein was quantified by Elisa and referred to untreated control cell cultures. No cytotoxicity of amantadine was observed until 100 µM. Remdesivir showed no cytotoxic effects in the tested concentration range. Results are mean ± SD of four (amantadine) or three (remdesivir) experiments; MOI, multiplicity of infection; IC_50_, half-maximal inhibitory concentration; * *p* < 0.05 compared to control cell cultures without drug exposition.

To test for the inhibitory potency of amantadine on replication of SARS-CoV-2, viral nucleic acids in the cytosol and in the cell culture medium were determined. Amantadine itself had no cytotoxic effect up to 100 µM in the medium (Figure 2). Some cytotoxicity was observed in the cell monolayer of uninfected cells at the highest concentration of amantadine (28% at 500 μM; see Figure 2A,C) only, and a cytoprotective effect of amantadine was not observed. Remdesivir as the comparative antiviral substance had no cytotoxic effects in the tested concentration range.

Amantadine 10–500 µM caused a concentration-dependent reduction (IC_50_ = 83 µM) of viral nucleic acids in the supernatant 26 h after infection (Figure 2A).

In addition, in these cells intracellular viral nucleic acids were determined. Amantadine 10–500 µM caused a concentration-dependent reduction (IC_50_ = 119 µM) of viral nucleic acids in the cytosol 26 h after infection (Figure 2C).

In a second series of experiments using a nucleocapsid protein ELISA for readout instead of qPCR amantadine, 30–300 µM caused a concentration-dependent reduction (IC_50_ = 88 µM) of viral nucleocapsid protein in the supernatant 72 h after infection (Figure 2E). Remdesivir activity was studied for comparison in all experiments (Figure 2B,D,F) and resulted in the low micromolar range (IC_50_ = 1.9–3 µM), in line with previous data [31]. No cytoprotective effect of amantadine was observed in the infected cell monolayers.

## 4. Discussion

The present experiments demonstrate an inhibitory effect of amantadine on SARS-CoV-2 infected Vero E6 cells at an IC_50_ between 83 and 119 µM. Interference with binding of the viral S-protein to ACE2 on target cells was neither observed for amantadine nor for remdesivir and is apparently not an important part of their mode of infection. The data has to be seen with caution because there is no good positive control for the binding step. Binding of the viral S-protein to ACE2 was also not observed with camostat mesylate, which is in line with its inhibitory effect on the human serine protease TMPRSS2 [32]. So, camostat mesylate does inhibit the virus entry but does not interfere with the binding to ACE2.

In patients receiving regularly 600 mg amantadine orally per day, plasma concentrations of up to 14.6 µM were found [33]. Higher doses were not used for any indication. After inhalation of amantadine 1 g/100 mL twice a day for 6 days a plasma concentration of 0.13 µM was found, whereas the concentrations in a nasal swab taken 60 min after inhalation contained 486 µM [34]. Side effects, mostly hallucinations or other psychotic symptoms, have been observed at plasma concentrations exceeding about 6 µM. Albrektson [35] reported an amantadine intoxication case with encephalopathy, agitation, respiration problems, and hypoxia, but no renal function impairment after intake of 1000 mg/day resulting in serum concentrations of 15.9 µM. Thus, the concentration required for inhibition of SARS-CoV-2 replication in Vero E6 cells is 5–8 times higher than the plasma concentrations observed in patients after therapeutic doses (200–600 mg/day per os), which indicates that amantadine does not offer a therapeutic window between antiviral efficacy in vitro and systemic toxicity in vivo. As a result, oral or i.v. administration of high amantadine doses for COVID-19 treatment appear to be obsolete. The required inhibitory concentration may be achievable by topical administration into the nose or by inhalation, an administration method considered for influenza A therapy [34]. This could be a potential approach because the infection of human airways by SARS-CoV-2 does not occur homogeneously. The susceptibility of the airway epithelium to SARS-CoV-2 infection shows a gradient from highest in nasal epithelium to lowest in distal pulmonary epithelium [36]. The authors suggest that the nasal cavity is the initial site of infection and lung infection could be mediated by aspiration of high-titer virus secretions from the nasopharyngeal region. If this turns out to be the major infection mechanism keeping viral titers low in the nose secretion could be a useful strategy. It may be achieved by nose flushing with acid-buffered saline, hypertonic saline, antiseptic solutions such as ethanol or isopropanol, or antiviral solutions such as type 1 interferon [37]. There is not much evidence for these approaches yet, but they stimulate the idea that amantadine could be administered intranasally or by aerosol inhalation. Inhalational amantadine therapy for influenza A was never officially approved but offered an attractive administration method as long as amantadine was effective. The amantadine safety of inhalative administration has been studied in healthy adult volunteers [34]; the authors had the volunteers inhale amantadine 1–2.5 g/100 mL 2x per day for 30 min each over 6 days. The amantadine concentration in the nasal wash 60 min after completion of inhalation of the 1% solution was 486 µM, while the plasma concentration at the same time was not detectable; after 120 min it reached 0.11 µM. The only observed physiological effect was a 12% reduction of maximal expiratory flow rate after 3 or 7 days repeated aerosol treatment, which returned to baseline after treatment termination. This effect was not concentration-dependent and could not be attributed to amantadine or to the aerosol inhalation itself. It was concluded that the small particle aerosol treatment with amantadine could be safely administered. Currently, the only marketed liquid formulation of amantadine is a 1 mM amantadine hemisulfate (400 mg/L) for intravenous infusion, which contains a slightly lower concentration than administered by Hayden.

Other cell lines may be better suited as a model for human airway epithelial infection than Vero E6 cells, which makes a transfer of the experimental data to human trials difficult. It has been shown in the example of chloroquine that other cell lines may better reflect the SARS-CoV-2 mode of infection and replication in human cells [38]. Hence, a repetition of the experiments in other cells, for example, the human lung cell line Calu-3, should be performed prior to any further investigations or clinical tests. A therapeutic window after systemic administration of amantadine cannot be concluded because the in vitro inhibitory concentrations were higher than safe plasma concentrations; sufficient concentrations may only be achievable after topical administration. Virus replication-inhibitory concentrations in vitro can hardly be directly compared to human plasma concentrations; however, this transfer is the first step of efficacy testing. The mode of action of amantadine on SARS-CoV-2 remains to be elucidated.

## 5. Conclusions

In conclusion, amantadine inhibits viral replication in the Vero E6 cell system. In this study, a functionally relevant interference with the binding of the viral spike protein to ACE2 on target cells could not be shown, with the limitations discussed above. The question was triggered by the predicted docking with close contact of amantadine to Tyr489 and Phe456 in the receptor-binding domain (RBD) of SARS-CoV-2 [39]; SARS-CoV-2 RBD (residues Arg319–Phe541) interacts with the N-terminal peptidase domain of ACE2 (residues Ser19–Asp615), which might have indicated a potential antiviral mode of action of amantadine [40], but our data do not substantiate the in silico hypothesis. Inhibition of a viroporin as an alternative mode of action needs to be analyzed in future studies. In a recently published preprint, amantadine inhibited the recombinant SARS-CoV-2 viroporin protein E and the putative SARS-CoV-2 viroporin Orf10 [16]. The authors observed in the Xenopus laevis oocyte model a 77% inhibition of the protein E ion channel-mediated current at 10 µM amantadine, which appears even more potent than the inhibition of the overall virus replication in the more complex eukaryotic cell culture model at an IC_50_ of 83–119 µM that we have found; these data indicate that viroporin inhibitors merit a closer look. Finally, so far, amantadine appears to also affect the known SARS-CoV-2 mutations because few or no mutations have been identified in protein E or Orf10 in mutated SARS-CoV-2 lineages collected from patients in India [41]. Lineage B 1.1.7 neither contains mutations in protein E nor in Orf10 [42]. Nevertheless, a single amino acid exchange can reduce the efficacy of a small molecule, as happened with the influenza A virus years ago [26].
